# Novel large-scale chromosomal transfer in *Bacteroides fragilis* contributes to its pan-genome and rapid environmental adaptation

**DOI:** 10.1099/mgen.0.000136

**Published:** 2017-11-14

**Authors:** Fasahath Husain, Kevin Tang, Yaligara Veeranagouda, Renata Boente, Sheila Patrick, Garry Blakely, Hannah M. Wexler

**Affiliations:** ^1^​ Brentwood Biomedical Research Institute, Los Angeles, CA, USA; ^2^​ UCLA, Los Angeles, CA, USA; ^3^​ GLAVAHCS, Los Angeles, CA, USA; ^4^​ Queens University Belfast, Belfast, UK; ^5^​ University of Edinburgh, Edinburgh, UK; ^6^​ Research, GLAVAHCS, 11301 Wilshire Blvd., 691/151J Bldg. 115, Room 312, Los Angeles, CA, USA

**Keywords:** *Bacteroides fragilis*, horizontal transmission, restriction modification system, polysaccharide capsules

## Abstract

*Bacteroides fragilis*, an important component of the human gastrointestinal microbiota, can cause lethal extra-intestinal infection upon escape from the gastrointestinal tract. We demonstrated transfer and recombination of large chromosomal segments from *B. fragilis* HMW615, a multidrug resistant clinical isolate, to *B. fragilis* 638R. In one example, the transfer of a segment of ~435 Kb/356 genes replaced ~413 Kb/326 genes of the *B. fragilis* 638R chromosome. In addition to transfer of antibiotic resistance genes, these transfers (1) replaced complete divergent polysaccharide biosynthesis loci; (2) replaced DNA inversion-controlled intergenic shufflons (that control expression of genes encoding starch utilization system outer membrane proteins) with more complex, divergent shufflons; and (3) introduced additional intergenic shufflons encoding divergent Type 1 restriction/modification systems. Conjugative transposon-like genes within a transferred segment and within a putative integrative conjugative element (ICE5) ~45 kb downstream from the transferred segment both encode proteins that may be involved in the observed transfer. These data indicate that chromosomal transfer is a driver of antigenic diversity and nutrient adaptation in *Bacteroides* that (1) contributes to the dissemination of the extensive *B. fragilis* pan-genome, (2) allows rapid adaptation to a changing environment and (3) can confer pathogenic characteristics to host symbionts.

## Abbreviations

BF, Bacteroides fragilis; CTn, Conjugative Transposon; HGT, Horizontal Gene Transfer; ICE, Integrative Conjugative Element; MGE, Mobile Genetic Element; ori, origin of replication; oriT, origin of transfer; PS, Polysaccharide; R-M, Restriction-Modification.

## Data Summary

All supporting data, code and protocols have been provided within the article or through supplementary data files.

## Impact Statement

*Bacteroides fragilis* has a formidable pan-genome which facilitates adaptation to distinct ecological niches, interactions with the host immune system within the GI tract and also enables the transition of the bacterium from symbiont to pathogen. The novel horizontal gene transfer (HGT) event reported here resulted in the insertion of the largest segment of *Bacteroides* chromosomal DNA ever reported and effected changes in the polysaccharide loci profile, control of nutrient binding systems and phage defense systems. The scale and mechanism of this transfer and potential phenotypic impact has not been demonstrated before and represents a mechanism for dissemination of the *Bacteroides* pan-genome as well as rapid adaptation to a changing host environment.

Transfer of these chromosomal segments (1) replaced either one (in the case of *tetQ2*_435_ and *tetQ2*_356_) or two (in the case of *tetQ2*_482_) entire micro-capsule operon(s) in *B. fragilis* 638R with homologous but divergent micro-capsule operons from *B. fragilis* HMW615, (2) replaced a shufflon that controls nutrient binding and outer membrane protein expression with a divergent and more complex shufflon and (3) introduced a second divergent R-M system gene shufflon, thus contributing to the remarkable redundancy that is characteristic of the *B. fragilis* genome.

## Introduction

*Bacteroides fragilis*, a Gram-negative anaerobic rod, is a commensal in the human gut [1] that can cause severe infections when it escapes its niche [2]. One of the remarkable traits of *B. fragilis* is its genetic plasticity, due in part to frequent genetic rearrangements; these include inversions, duplications and horizontal gene transfer (HGT) mediated by a variety of mobile and mobilizable elements [3]. This genetic plasticity facilitates adaptation to distinct ecological niches [3–5] and enhances the spread of antibiotic resistance determinants [6, 7]. Another notable trait of *B. fragilis* is the extensive redundancy within its genome, including the wide variety of carbohydrate utilization systems that provide factors important both for colonization and energy metabolism [8, 9].

HGT is regarded as a major mechanism of prokaryotic evolution [10–15]; the scale of its importance has been more clearly delineated in recent years. HGT can occur by transformation (acquisition of DNA from the local environment), by phage-mediated transduction and by conjugation via cell-to-cell contact [16–19]. After transfer, the chromosomal segments can integrate into the recipient chromosome through either site-specific or homologous recombination [20]. The nomenclature of these horizontally transferred elements is currently in flux. The most inclusive term, Genomic Islands (GIs), describe HGT-acquired gene clusters that are often associated with adaptations to the niche of the organism and that contribute to evolutionary change [21]. GIs include the group described as integrative and conjugative elements (ICEs) which in turn includes the group of elements described as conjugative transposons (CTns) [17, 22].

In *Bacteroides* species, integrative and conjugative elements (ICE), including conjugative transposons (CTns), are considered important mediators of horizontal gene transfer (HGT) [23]. CTnDOT, for example, is responsible for transfer of tetracycline resistance. We recently reported a novel *Bacteroides* CTn, CTnHyb (isolated from *B. fragilis* HMW615) [24]. CTnHyb is a mosaic of mobile elements from Gram-positive bacteria and transferred tetracycline, kanamycin and metronidazole resistance genes as well as efflux pump genes that may also be associated with antibiotic resistance in *B. fragilis* [24, 25]. Remarkably, the extensive horizontal gene transfer among *Bacteroides* and apparent incorporation of genes from other genera occurs despite the diverse DNA restriction and modification systems in *Bacteroides* genomes. The presence of genes encoding homologues of the anti-restriction protein, ArdA, has been noted within some CTns. These proteins mimic B-form DNA and block binding of Type 1 R-M systems [26] and might help to explain why these elements can be transferred successfully between divergent strains.

The plasticity and ability of *B. fragilis* to adapt to various niches is due, in part, to modulation of surface antigenicity through variable production of multiple different surface proteins and capsular polysaccharides. Ten or more polysaccharide biosynthesis-associated loci may be present within an individual genome and at least eight of these are associated with production of a microcapsule; the genetic loci encoding these genes can be turned on or off by inversion of promoter sequences [27]. In addition, microcapsule expression is controlled by multiple transcriptional anti-terminators [28]; regulation of these multiple microcapsules is important for colonization of *Bacteroides* in the host [29, 30]. *B. fragilis* also exhibits an unprecedented between-strain antigenic diversity of polysaccharide expression as a result of extensive diversity of PS biosynthesis loci among strains; 28 different PS biosynthesis associated loci were identified in just three isolates [28]. This suggests that *B. fragilis* has a pan-genome with an extensive pool of polysaccharide biosynthesis-associated genes.

The mode of generating polysaccharide diversity between and amongst strains has not been clarified. CTns have been reported to be associated with PS biosynthesis locus duplication within a *B. vulgatus* genome [31] and CTn and bacteriophage insertion have been observed to disrupt PS synthesis loci [28, 31]. However, the precise mechanism(s) generating the HGT underpinning the observed diversity of PS synthesis loci has, to date, been open to speculation. We show here that large scale chromosomal transfer, likely as a result of conjugation and integration via homologous recombination, mediated replacement of a complete PS biosynthesis region with a divergent PS biosynthesis region, replaced an intergenic shufflon which controls variable transcription of genes encoding outer membrane proteins, and introduced a second shufflon encoding a variable Type 1 restriction and modification (R-M) systems. This transfer helps to explain the remarkable redundancy present in the *B. fragilis* genome and demonstrates a mechanism for transmission of the pan-genome.

## Methods

### Bacterial strains and culture conditions

*B. fragilis* 638R [32] is a plasmid-free, rifampicin resistant *B. fragilis* that is frequently used for molecular manipulations; rifampicin is used to select against the donor strain in mating experiments. *B. fragilis* HMW615 is a multidrug resistant clinical isolate from an appendiceal abscess [33]. *B. fragilis* type strain NCTC 9343 (ATCC 25285) was used as the control for the MIC determinations. All strains of *B. fragilis* were grown anaerobically (5 % carbon dioxide, 5 % hydrogen, 90 % nitrogen) overnight in supplemented Brain Heart Infusion (BHIS) broth or Agar (Anaerobe systems, CA) at 37 °C. Antibiotics (including rifampicin 10 µg ml^−1^ or/and tetracycline 1 µg ml^−1^ [Sigma-Aldrich]) were prepared as directed and used as needed. Prepared media was purchased from Anaerobe Systems.

### Mating of *B. fragilis* 638R and *B. fragilis* HMW615

*B. fragilis* 638R and *B. fragilis* HMW615 were grown anaerobically overnight at 37 °C in BHIS and the mating conducted by described methods [24]. Only one colony from each mating plate was chosen for further analysis to minimize detection of sibling colonies.

To test the effects of tetracycline on transfer rates, *B. fragilis* HMW615 was either grown overnight in the presence of tetracycline (1 µg ml^−1^) or grown without antibiotic and then treated with tetracycline (1 µg ml^−1^) for 1 h at 37 °C. Treated cells were then washed twice with equal volumes of BHIS and the regular mating procedure with *B. fragilis* 638R was followed. In an alternative method to test effects of tetracycline on mating frequency, *B. fragilis* 638R and *B. fragilis* HMW615 were grown separately overnight and 200 µl of each were mixed and plated on BHIS containing 0.001 µg ml^−1^ of tetracycline, and incubated anaerobically overnight at 37 °C before mating [24].

### Molecular techniques

Genomic DNA for PCR analyses was prepared using the DNeasy Blood and Tissue kit (Qiagen). One Taq Polymerase master mix (NEB) was used for the PCR procedures. Amplicons were sequenced using Sanger sequencing (Laragen, Culver City, CA). The primers used are listed in Table S1 (available in the online Supplementary Material).

### Genome sequencing of recombinant strains and comparative genome analysis

Genomic DNA was prepared using the Qiagen DNeasy Blood and Tissue Kit and submitted for Mi-Seq analysis (Laragen). The data were analyzed using the DNASTAR Lasergene Genomics Suite (DNASTAR). FastQ files were assembled with the SeqMan NGen module using *B. fragilis* 638R as the template sequence (638R_NC016776.1; GI : 375356399) and the default parameters. Comparisons of the aligned files were visualized using the SeqMan Pro module and areas of conflict are shown. Detection of prophage sequences within the genome was done with the Phaster tool [34, 35].

### DNA alignment and gene comparison

*B. fragilis* HMW615 was sequenced as part of the Human Microbiome Project, *Bacteroides* group Sequencing Project, Broad Institute of Harvard and MIT (http://www.broadinstitute.org/) and was given the designation HMPREF1204. *B. fragilis* HMW615 genes are referred to by HMPREF1204 locus tags which were assigned by the Broad Institute prior to final genome assembly.

The *B. fragilis* 638R and *B. fragilis* HMW615 genome sequences were acquired as GenBank files from the NCBI server [36]. The RAST server was used to identify homologs between genomes [37]. Genome comparisons were done using the Double ACT server (http://www.hpa-bioinfotools.org.uk) and viewed with the Artemis Comparison Tool (ACT) viewer [38]. When needed, nucleotide or amino acid sequences were aligned using ClustalW. Based on our genome analysis, we realigned the *B. fragilis* HMW615 genome using the MAUVE alignment tool [39] and used the predicted origin of replication site (*ori*) to generate an updated GenBank file which was then used for our subsequent comparative genome analyses with the Artemis and ACT tools [38]. Circular representation of the genome was generated using DNAPlotter [40].

## Results and discussion

### Mating of the clinical multidrug resistant isolate *B. fragilis* HMW615 with *B. fragilis* 638R resulted in the transfer of DNA segments of varying sizes (125 Kb to 482 Kb) from *B. fragilis* HMW615 to *B. fragilis* 638R

Originally, twenty independent matings were carried out as described [24] with subsequent selection for rifampicin and tetracycline resistance; 5–10 colonies were isolated on each plate. To reduce the likelihood of detecting siblings, only one colony was chosen from each plate for further analysis. Several individual isolates were chosen for further analysis. *B. fragilis* HMW615 encodes two *tetQ* paralogues [HMPREF1204_02983 (*tetQ1)* and HMPREF1204_03369 (*tetQ2)*]. Sequencing of the region surrounding the respective *tetQ* genes in the individual isolates revealed that separate events had occurred, each resulting in one of the two *tetQ* paralogues being transferred. *tetQ1* was contained within a genomic segment that we recently identified as a novel conjugative transposon, CTnHyb, composed of *B. fragilis* genes as well as a mosaic of genes from various aerobic genera, including several genes encoding resistance determinants [24]. *tetQ2* is in a region of the HMW615 chromosome with extensive homology to *B. fragilis* 638R. We found that transfer of *tetQ1* (i.e., the transfer of CTnHyb) was 10X more frequent than the transfer of *tetQ2* described in this report. As previously estimated, the frequency of the transfer was 1×10^−9^ (number of transconjugants [BF638R-CtnHyb]/numbers of donors [BF HMW615]) for *tetQ1*. Among the 200 to 500 colonies that were seen in each of the mating reactions, approximately every 10th colony was *tetQ2* (the others were *tetQ1*).

The recombinants identified and analyzed in this report are referred to as BF638R :: *tetQ2*-_125 to 482_, to reflect the host, the transferred *tetQ2* gene and the size of the chromosomal segment that was transferred and integrated into the host genome (the respective chromosomal segments are referred to as *tetQ2*-_125 to 482_). We confirmed that the recombinants bearing *tetQ2* were of *B. fragilis* 638R origin. This confirmation was required because tetracycline-resistant, rifampicin-resistant colonies isolated from the mating mix could have arisen as a result of (1) spontaneous mutation of the *rpoC* RNA polymerase beta subunit gene (conferring rifampicin resistance) in *B. fragilis* HMW615 or (2) transfer of the mutated *rpoC* [28] from *B. fragilis* 638R to *B. fragilis* HMW615. PCR analysis and sequencing of the recombinants confirmed the presence of BF638R_2089 and BF638R_4382 – two genes with no homologues in *B. fragilis* HMW615 – thus confirming their *B. fragilis* 638R origin. Subsequent complete genomic sequencing of the recombinants further confirmed these results.

### The mechanism of transfer of the *tetQ2*-chromosomal segments is not known

**Fig. 1.**
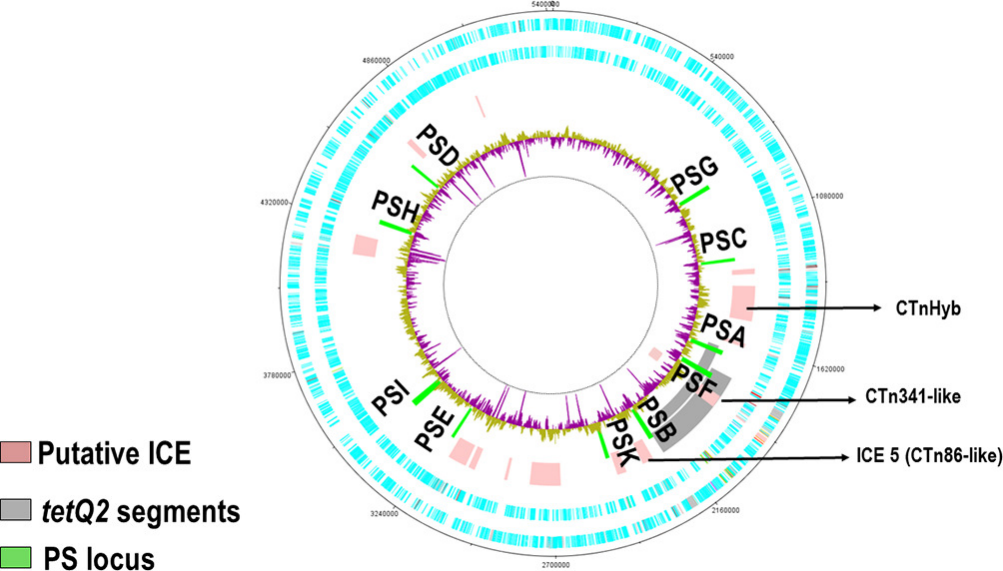
Circular representation of the *B. fragilis* HMW615 chromosome illustrates the position of *tetQ2*_435_, other putative ICEs and polysaccharide (PS) associated loci. From the outside: Circle 1, DNA coordinates; Circle 2 and 3, CDs forward/reverse strands; Circle 4, putative integrative and conjugative elements (ICE) including *tetQ2*_435_ (Note: *tetQ2*_435_ is marked in this figure as an example of the *tetQ2* segment positions); Circle 5, surface polysaccharide synthesis associated loci (PSA-K); Circle 6, graph of %GC content. Above genome average %GC (yellow/green), below average (purple). Note that graph spikes reaching the inner circle relate to %GC sequence assembly gaps.

Conjugation is, however, the most likely mechanism of transfer as: (1) there is no evidence that *B. fragilis* is naturally competent for uptake of DNA (even transformation via electroporation is often inefficient); (2) *B. fragilis* HMW 615 is plasmid free and thus plasmid-mediated transfer is ruled out; and (3) *B. fragilis* HMW 615 does not encode a phage genome that could result in transduction. Fig. 1 is a circular representation of the *B. fragilis* HMW615 chromosome that illustrates the position of the transferred segments within recombinants BF 638R :: *tetQ2*_435_ and BF 638R :: *tetQ2*_482_. We speculate that co-resident CTns, the CTn341-like region within the transferred segment and/or the CTn86-like ICE5 (49.5 kb downstream of the end of *tetQ2*_435_), in *B. fragilis* HMW 615 provided the functions required for transfer.

### The transfer of DNA in the *tetQ2*-chromosomal segments is consistent with an excision-independent mechanism

Since the lengths of DNA transferred were variable, we believe it likely that the transfer of these *tetQ2*-containing segments did not include excision from the chromosome prior to conjugation. Excision-independent transfers have been described in both Gram-negative and Gram-positive bacteria, with up to 1 Mb of chromosomal DNA being transferred [41, 42]. Transfer of a *B. fragilis* chromosomal segment mediated by one of the resident elements, such as ICE5, in *B. fragilis* HMW615 would be consistent with these previous observations. For example, an *oriT* from the downstream ICE5 could be responsible for cleavage and transfer in an Hfr-like mechanism.

Another CTn-related Hfr-type transfer was described in a *Bacteroides* strain carrying an excision deficient version of conjugative transposon CTnERL which contained an insertion in the integrase gene [43]. Normal CTnERL excision and integration are independent of homologous recombination. In the case of the integrase deficient CTnERL, however, homologous recombination was needed to ‘rescue’ the segment of CTnERL DNA that had transferred. The authors noted that these were the first studies to show that transfer of a portion of the CTn is possible and that the transferred DNA can be rescued if there is a closely related CTn present in the recipient chromosome. They did not specifically measure the length of DNA transferred, because their studies identified transfer by using a selection marker that was only 20 kb from the *oriT* region.

### Mechanism of integration

**Fig. 2.**
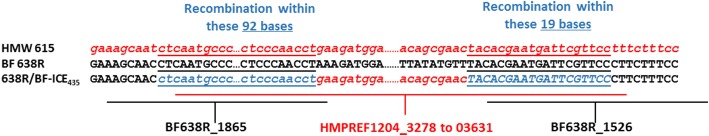
The recombination of regions of *B. fragilis* HMW615 and *B. fragilis* 638R that generated BF638R :: *tetQ2*_435_. The italicized (lowercase) sequence is *B. fragilis* HMW615 and the bold is *B. fragilis* 638R. Single nucleotide polymorphisms between the *B. fragilis* 638R and *B. fragilis* HMW615 genomes allowed localization of the recombination site to small regions, as shown. On the left side, strand exchange within the underlined 92 bp region produces a hybrid gene between BF638R_1526 and HMPREF1204_03278, which has 99 % aa identity to BF638R_1526 and 100 % aa identity to HMPREF1204_03278. On the right side, recombination occurs within the underlined 19 bp region and the hybrid gene created is 100 % homologous to HMPREF1204_03631.

We demonstrated previously that a conjugative transposon (CTnHyb) bearing *tetQ1* integrated through site-specific recombination [24]. Analysis of our initial recombinant bearing *tetQ2*, however, indicated that a large, 435 K bp DNA segment had transferred from *B. fragilis* HMW615 and replaced a 413 K bp sequence on the *B. fragilis* 638R chromosome. Subsequently, we isolated three additional recombinants carrying *tetQ2*. The segments were apparently integrated into the host genome via homologous recombination rather than site specific integration. PCR analysis of the left and right ends of the insertion into *B. fragilis* 638R :: *tetQ2*_435_ delineated the sites of recombination (Fig. 2). On the left end, the recombination occurred between BF638R_1526 and HMPREF1204_03278 and could be localized to a 92 bp region. BF638R_1526 and HMPREF1204_03278 (predicted outer membrane proteins) differ in nine bases, but have only a single amino acid change (V487I change in BF638R_1526). Recombination on the right end occurred between HMPREF1204_03632 and BF638R_1865 (Fig. 2). The 19 bases in which recombination occurred include the start codon of HMPREF1204_03632 (Fig. 2) and the RAST-assigned BF638R_1865 start codon.

**Fig. 3.**
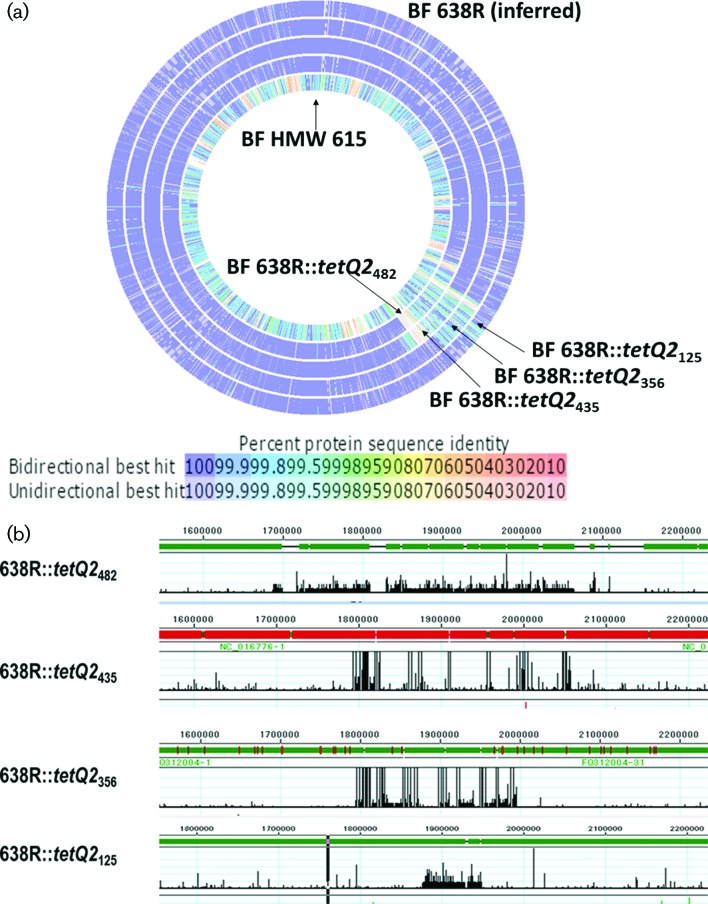
Genomic sequence comparison of *B. fragilis* 638R, *B. fragilis* HMW615 and the recombinants. (a) Comparison of the *B. fragilis* 638R sequence with the four recombinants (*B. fragilis* 638R *:: tetQ2*s) and *B. fragilis* HMW615 using RAST (http://rast.nmpdr.org/). Percent protein sequence identity is indicated by colour of the corresponding gene hit (legend). (b) Comparison after sequencing using MiSeq analysis and assembly with SeqMan Ngen using the *B. fragilis* 638R sequence as a reference and viewed in SeqMan Pro. The vertical black lines represent conflicts with the reference sequence. The width of the conflict area represents the length of the inserted sequence. As is apparent, there are areas within the insertion where the recombinants are homologous to the *B. fragilis* 638R sequence.

Subsequent whole genome sequence analysis of four recombinants was performed and the edges of the transferred segment and recombination points in *B. fragilis* 638R were identified. The lengths of the transferred segments ranged from 125 to 482 K bp (Table 1). Fig. 3(a) provides an overall view of the area of insertion into the *B. fragilis* 638R chromosome, degree of homology between the donor and host genomes and the recombinants resulting from the events described. Fig. 3(b) is a representation of the areas of differing sequence between the recombinants and the *B. fragilis* 638R genome. The width of the conflict area represents the length of the inserted segment. The genes transferred by the two smaller chromosomal segments, *tetQ2*_125_ and *tetQ*_356,_ were wholly contained within *tetQ*2_435_.

**Table 1.** Characteristics of *B. fragilis* (BF) 638R recombinants resulting from horizontal transfer of *tetQ2*-containing chromosomal segments

**Strain**	**Size of 638R segment replaced**	**Size of HMW615 segment transferred**	**638R Left end boundary/Locus Tags**	**615 Left end boundary/Locus Tags**	**615 Right end boundary/Locus Tags**	**638R Right end boundary/Locus Tags**
BF638R :: *tetQ2*_435_	412498	435461	BF638R_1526	HMPREF1204_03278	HMPREF1204_03631, 03632	BF638R_1865
BF638R :: *tetQ2*_125_	70896	124549	BF638R_1604	HMPREF1204_03357	HMPREF1204_03467	BF638R_1665
BF638R :: *tetQ2*_356_	258992	359673	BF638R_1526	HMPREF1204_03278	HMPREF1204_03567	BF638R_1741
BF638R :: *tetQ2*_482_	418817	482533	BF638R_1421, 1422, tRNA	HMPREF1204_03179, 03180, tRNA	HMPREF1204_03584	BF638R_1785

The reciprocal exchange of loci seen in the recombinant, rather than insertion of the foreign DNA, indicates that recombination most likely occurred through RecA-dependent homologous recombination. In a possible scenario, the early events leading to acquisition of the *tetQ2*-containing chromosomal segment would have resembled the mechanism of CTn conjugation. In this scenario, B*. fragilis* HMW615 DNA was nicked and cleaved at the *oriT* within ICE5 (Fig. 1) and subsequently transferred into *B. fragilis* 638R. In the recipient, the incoming 5′ single-stranded DNA linked to the relaxosome complex was then replicated by lagging strand synthesis to generate double-stranded DNA. Since the AddAB helicase-nuclease can translocate and degrade DNA up to 45 kb from a double-strand end *in vitro* [44], it is plausible that exonuclease activity of AddAB could process this DNA segment (prior to RecA-mediated strand invasion), removing evidence of the ICE5 *oriT* in the recombinant. The 3′ RecA-loaded single strands would then allow duplex invasion in an ‘ends-out’ configuration [45]. This would be similar to the transfer of the pathogenicity locus of *C. difficile* to non-toxigenic strains. In that study, the pathogenicity locus (PaLoc) could be transferred from the toxin-producing strain to non-toxigenic strains and the recipient strains were positive in assays for cytotoxin B [46]. Because the PaLoc was not contained within a mobile element, the authors concluded that an *oriT* within the host mediated transfer and the incoming DNA was integrated into the recipient chromosome by homologous recombination. Further, like our variably sized segments, the PaLoc was transferred on variably sized DNA segments (66 034–272 977 bp).

### Gene substitutions, additions, deletions or duplications caused by integration of the *tetQ2*-containing chromosomal segments in *B. fragilis* 638R

**Fig. 4.**
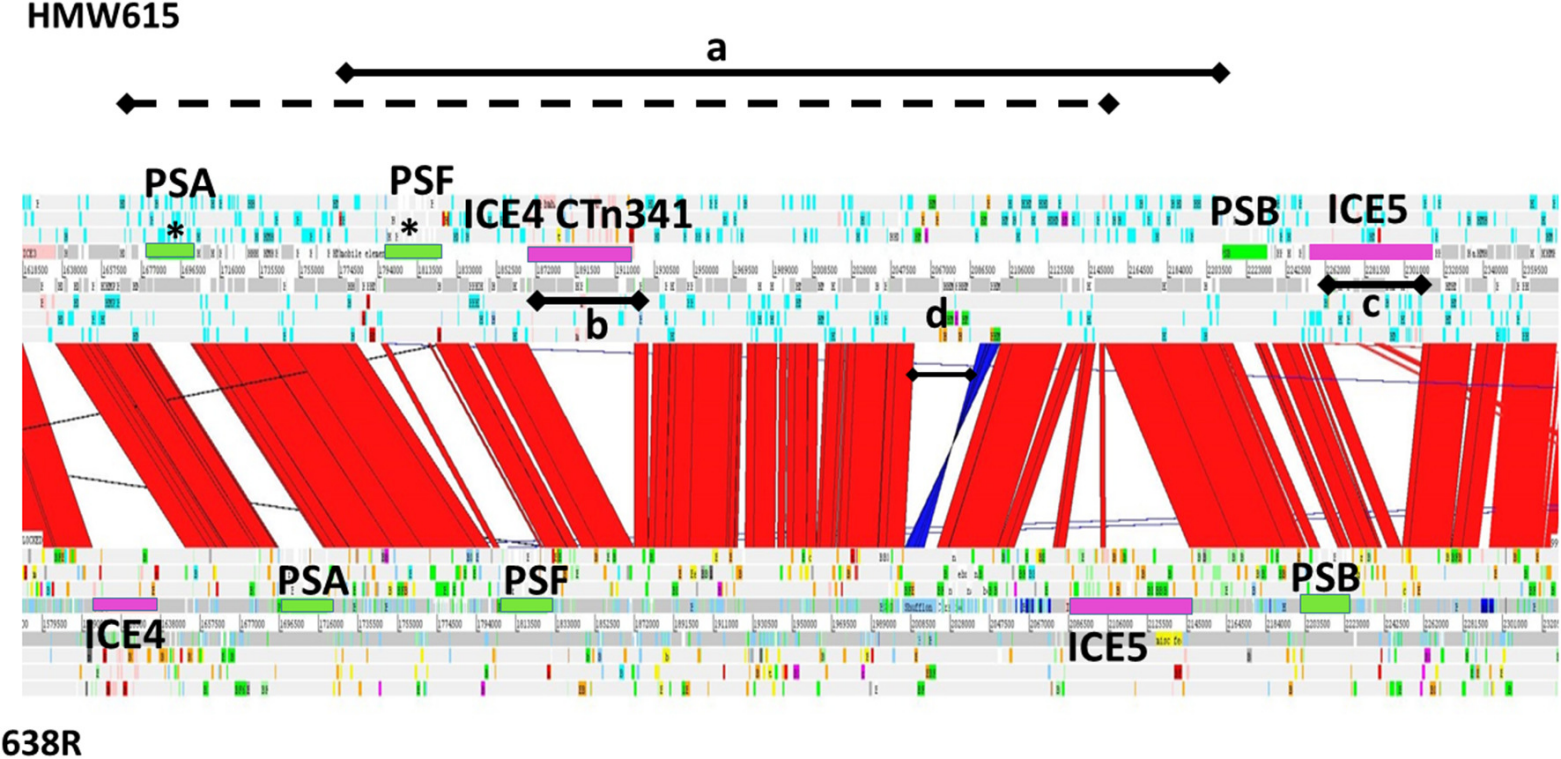
Artemis Comparison Tool genome comparison illustrating transfer of novel polysaccharide biosynthesis loci. Red colouration between sequences indicates sequence identity, no colour indicates divergent sequence, dark blue indicates inverted sequence. PS biosynthesis associated loci, green; putative integrative and conjugative elements (ICE), light pink: a, BF638R *:: tetQ2*_435_ (solid line); BF638R *:: tetQ2*_435_ (dashed line) chromosomal segments transferred from HMW615 into 638R; b, CTn341-like element embedded within transferred chromosomal segments; c; CTn86-like element; d, Intergenic shufflon. Transfer of *tetQ2*_482_ has introduced two divergent micro-capsule polysaccharide biosynthesis operons, one at the PSA and one at the PSF locus (*).

The exact range of genes transferred in the *tetQ2*-containing chromosomal segments, as well as the genes replaced in *B. fragilis* 638R, are listed in Table 1. The DNA segments integrated ranged from 124 549 to 482 538 bp and replaced segments of the *B. fragilis* 638R chromosome ranging from 70 896 bp to 418 817 bp. Fig. 4 is an ACT comparison between *B. fragilis* HMW615 and *B. fragilis* 638R of the region encoding polysaccharide microcapsules and highlights several of the important locations in the two largest segments transferred (tetQ2_482_ and tetQ2_435_).

Tables S2 and S3 compare the gene content of *B. fragilis* 638R and the *B. fragilis* 638R :: *tetQ2* recombinants using comparative genome analysis by RAST [37]. For example, eighty-four novel genes were introduced from HMW615 to 638R and seventy-nine genes were lost from 638R by the transfer and subsequent recombination of *tetQ2*_435_ to *B. fragilis* 638R.

### Integration of the *tetQ2*s_356, 435, and 482_ chromosomal segments replaced the *B. fragilis* 638R PSF micro-capsule locus with a homologous, but not identical, PSF micro-capsule operon

**Fig. 5.**
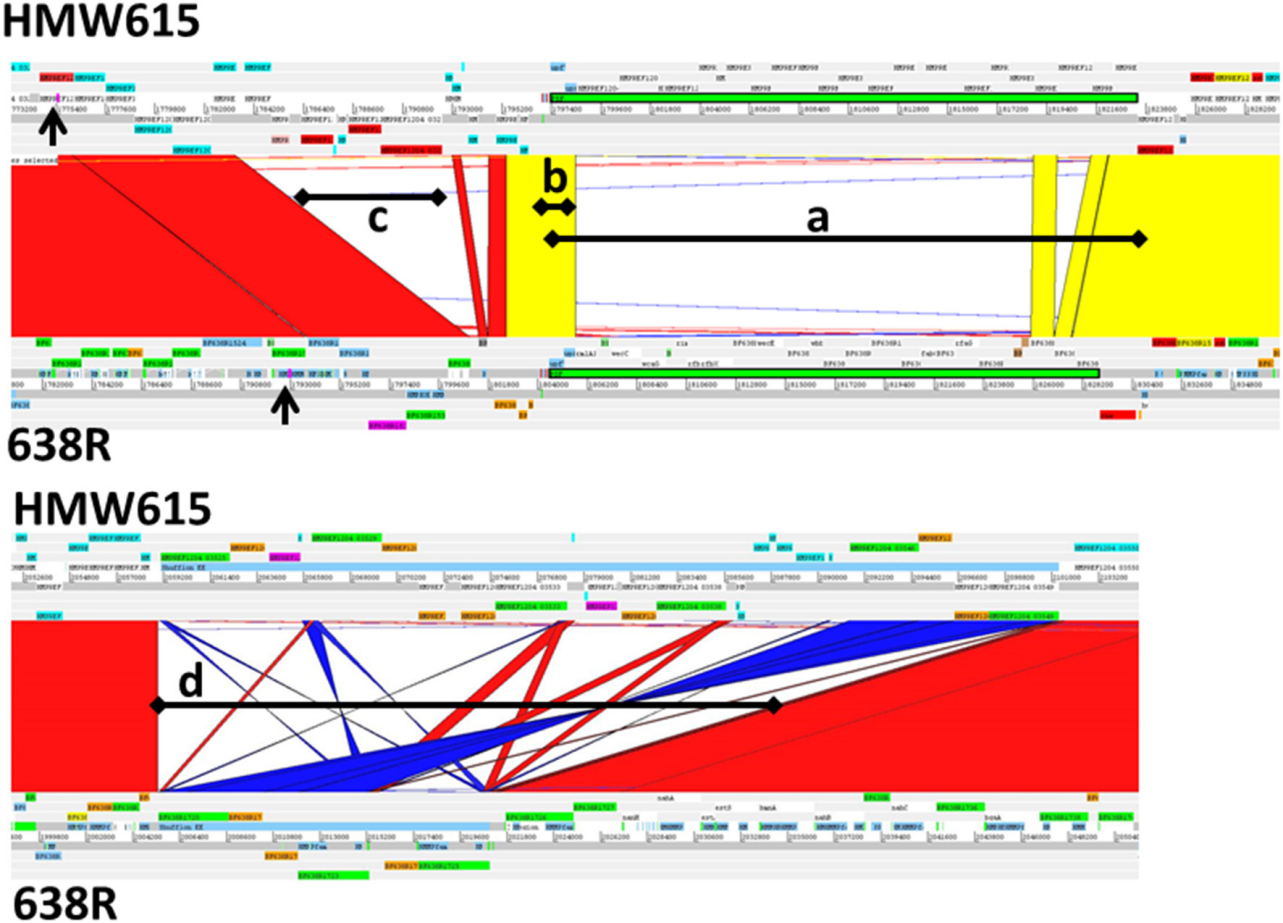
Genome comparison detail of selected regions within *tetQ2*_435_ using the Artemis Comparison Tool. Red/yellow colouration between sequences indicates sequence identity, no colour indicates divergent sequence, dark blue inverted sequence. Left, *tetQ2*_435_ recombination region, arrows. Upper panel: (a), Divergent microcapsule PSF biosynthesis associated locus; (b), Conserved PSF regulatory genes and invertible promoter; (c), Type I restriction and modification genes absent in *B. fragilis* 638R Lower panel: (d), Intergenic shufflon with invertible DNA regions encoding protein pairs of outer membrane associated proteins.

**Fig. 6.**
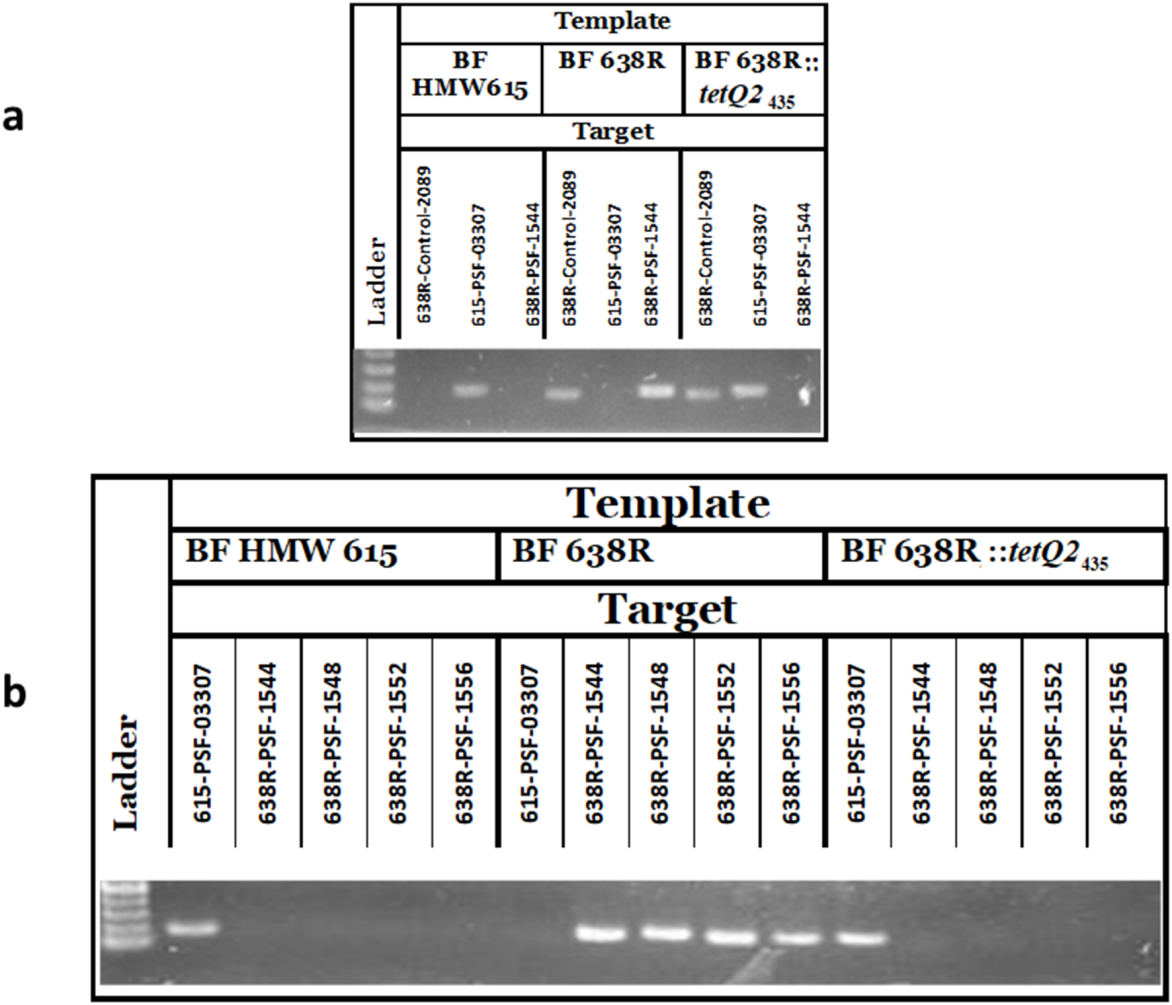
PSF exchange mediated by *tetQ2*_435_ and *tetQ2*_482._ HMPREF1204_03307, is a gene from the PSF operon of *B. fragilis* HMW615 (within *tetQ2*_435_). BF638R_1544, BF638R_1548, BF638R_1552, and BF638R_1556 are genes within the PSF operon of *B. fragilis* 638R. BF638R_2089 is a *B. fragilis* 638R specific gene outside the region replaced by *tetQ2*_435_ that is not present on the *B. fragilis* HMW615 genome. a: Lane 1: MW Standard, Lanes 2–6: Template *B. fragilis* HMW615. Primers for HMPREF1204_03307, BF638R_1544, BF638R_1548, BF638R_1552, and BF638R_1556, respectively. Lanes 7–11: Template BF 638R. Primers for HMPREF1204_03307, BF638R_1544, BF638R_1548, BF638R_1552, and BF638R_1556, respectively. Lanes 12–16: Template BF638R*::tetQ2*_435_. Primers for HMPREF1204_03307, BF638R_1544, BF638R_1548, BF638R_1552, and BF638R_1556, respectively. b: Lane 1: MW Standard. Lanes 2–4: Template *B. fragilis* HMW615. Primers for BF638R_2089, HMPREF1204_03307, BF638R_1544, respectively. Lanes 5–7: Template *B. fragilis* 638R. Primers for BF638R_2089, HMPREF1204_03307, BF638R_1544, respectively. Lanes 8–10: BF 638R *:: tetQ2*_435_. Primers for BF638R_2089, HMPREF1204_03307, BF638R_1544, respectively.

The variably produced and antigenically diverse polysaccharide micro-capsules of *B. fragilis* are extremely important elements implicated in both normal immune system development and abscess formation. Each *B. fragilis* strain studied to date contains multiple PS loci controlled by invertible promoters and transcriptional regulators. Comparison of three whole genome sequences revealed an extensive pool of 28 divergent PS biosynthesis loci [47]. Scrutiny of the *B. fragilis* HMW615 genome has added a further 5 divergent PS loci to the known *B. fragilis* pan-genome. A comparison of the PSF locus in *B. fragilis* HMW615 and *B. fragilis* 638R (Fig. 5, upper panel) shows conservation between the transcriptional regulators but a lack of DNA sequence identity within the polysaccharide synthesis associated genes. PCR analysis with primers specific for *B. fragilis* 638R PSF and *B. fragilis* HMW615 PSF and subsequent genomic sequencing confirmed that integration of *tetQ2*s_356, 435 and 482_ replaced the complete PSF locus of *B. fragilis* 638R with the divergent locus present in *B. fragilis* HMW615 which demonstrates one mechanism for generation of PS diversity; the results of the PCR analysis for *B. fragilis* 638R *:: tetQ2*_435_ are shown as an example in Fig. 6. Remarkably, in the longest transferred segment, *tetQ2*_482_, the entire PSA locus is also exchanged, in addition to the PSF locus described earlier. The downstream ICE 5 (Fig. 1) is not seen in any of the recombinants.

**Fig. 7.**
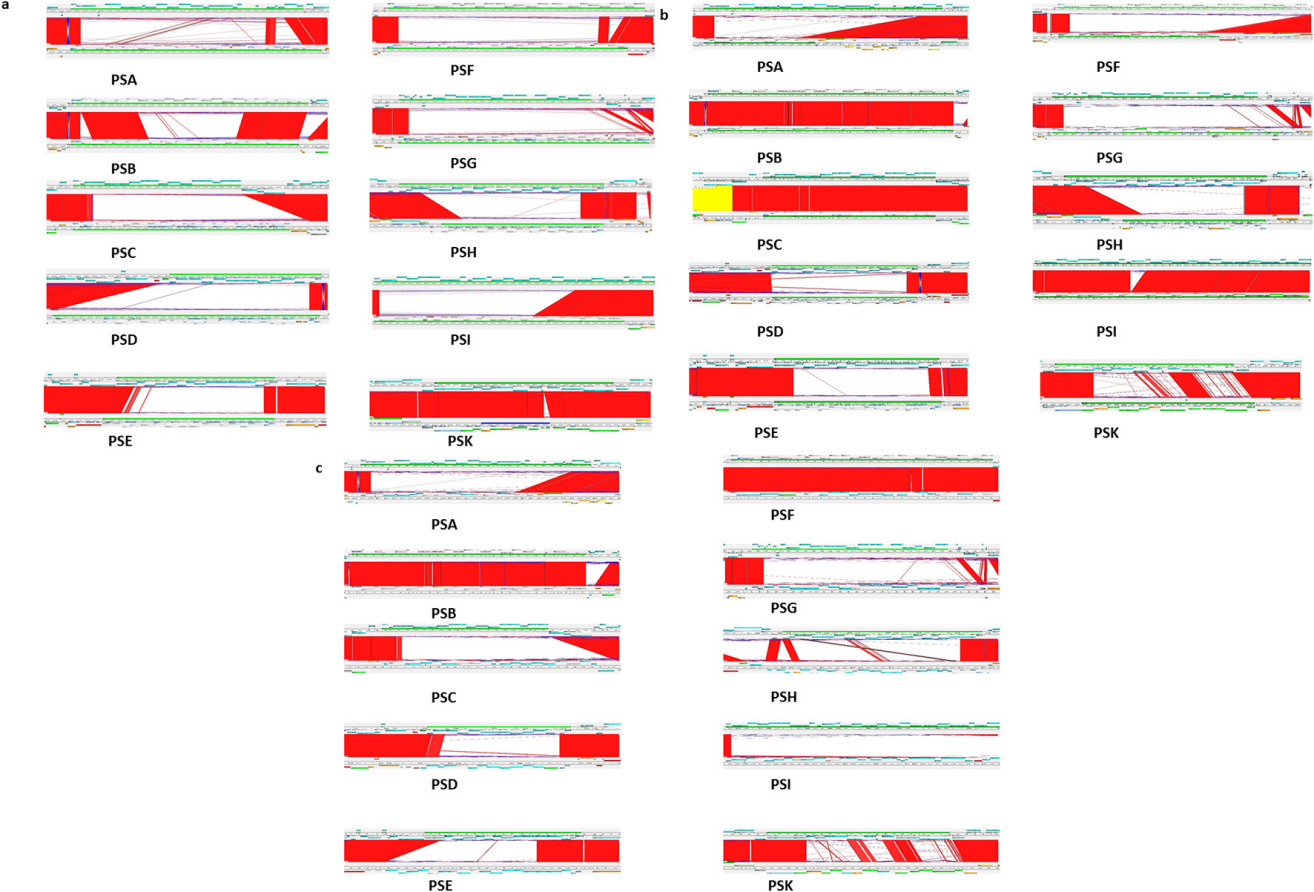
ACT comparison of PS loci of *B. fragilis* HMW 615 and *B. fragilis* 638R (7a), *B. fragilis* 9343 (7b) and *B. fragilis* YCH46 (7 c). Note gene conservation at the beginning and end of the PS operon. PSK was similar to the locus in *B. fragilis 638*R and only the loci for PSB, C, F and I were similar to loci seen in either *B. fragilis* ATCC 25285 (NCTC 9343) or YCH46.

Strain divergence in polysaccharide synthesis loci was seen in earlier comparisons of *B. fragilis* 638R, NCTC 9343 (ATCC 25285) and YCH46 [28]. ACT comparison of *B. fragilis* HMW615 with *B. fragilis* 638R, *B. fragilis* ATCC 25285 (NCTC 9343) and *B. fragilis* YCH46 (Fig. 7) revealed that PSK was similar to the locus in *B. fragilis 638*R (Fig. 7a) and that only the loci for PSB, C, F and I were similar to loci seen in either *B. fragilis* ATCC 25285 (NCTC 9343) (Fig. 7b) or YCH46 (Fig. 7c). Thus, *B. fragilis* HMW615 has five divergent PS biosynthesis loci (PSA, PSD, PSE, PSG, PSH) in addition to the 28 already described for *B. fragilis* 638R, *B. fragilis* NCTC9343 (ATCC25285) and *B. fragilis* YCH46, resulting in 33 divergent biosynthesis loci among the 4 strains. Interestingly, the genes within the PSB locus associated with the formation of a chemically stable carbon-phosphorus bond, *aep* X,Y and Z [48], are conserved although subsequent genes are divergent. PS locus sequence diversity is reflected by the production of micro-capsules of different antigenicity [28]. The mechanism underpinning this unprecedented level of diversity of PSs among *B. fragilis* was unknown although the difference in the %GC content from the core genome suggests horizontal gene transfer of these loci (Fig. 1).

The replacement of a complete PS biosynthesis locus (or loci) with a complete and divergent PS biosynthesis locus has revealed a mechanism for pan-genome dissemination of PS loci. Interestingly, the PSA of *B. fragilis* NCTC9343 (ATCC25285), which is linked to the down-regulation of the immune response within the gastro-intestinal tract [49], is not conserved in *B. fragilis* HMW615 or *B. fragilis* 638R. Which and how many of the divergent PSs within the *B. fragilis* pan-genome have similar properties to PSA remains to be determined, but transfer and replacement of PS loci associated with different types of immune system interaction could impact on the dynamics of immune system development. This in turn could alter the balance between immune tolerance and disease.

### The transfer of *tetQ2*_435_ replaced an intergenic shufflon which controls variable transcription of genes encoding outer membrane proteins

In *B. fragilis* NCTC 9343, there is a region overlapping the start codon of *ragA/susC* homologues that is composed of extensive inverted repeats (Region EE, intergenic shufflon) [28]; this entire region is downstream of an invertible promoter. In this system, inversion of large segments of DNA mediated by inverted repeats brings the alternative outer membrane protein genes downstream of this invertible promoter, thus enabling transcription of these genes. These regions encode pairs of genes similar to the *ragA/B* genes of *P. gingivalis* [50] and *susC/D* genes of *B. thetaiotaomicron* [51]. RagB is a major immunodominant outer membrane protein in *P. gingivalis* and SusC/D proteins are associated with nutrient binding at the cell surface. Here, *tetQ2*_435_ has replaced the equivalent *B. fragilis* 638R intergenic shufflon with another potentially invertible region containing divergent genes. Comparison of *B. fragilis* 638R and *B. fragilis* HMW615 indicates that the *tetQ2*_435_ transfer replaced the *B. fragilis* 638R shufflon containing three potential gene pairs with *B. fragilis* HMW615 shufflon which contains six genes, five of which are divergent (Fig. 5, lower panel). Thus, transfer of *tetQ2*_435_ has not only altered the PS surface antigenicity but also surface protein variability.

### The introduction of *tetQ2*_435_ introduced a second shufflon encoding a variable Type 1 restriction and modification (R-M) systems

Another highly variable feature of *B. fragilis* is the diversity of R-M systems, apparent both within a strain as a result of gene shuffling and also between strains. *B. fragilis* 638R has one R-M shufflon (BF638R 1146–1149). A second shufflon encoding four Type I R-M associated genes that are not present in *B. fragilis* 638R (HMPREF1204_03284, HMPREF1204_03286, HMPREF1204_03289 and HMPREF1204_03290) are evident at the left end of *tetQ2*_435_ (Fig. 5, upper panel, arrow c). HMPREF1204_03284 appears to be an S-subunit, so it could potentially replace HMPREF1204_03286 to generate an alternative DNA-binding specificity. An intervening integrase, HMPREF1204_03285, might be responsible for mediating site-specific recombination to rearrange the genes encoding the DNA-binding S-subunits. This second R-M modification locus was transferred to all but *B. fragilis* 638R *:: tetQ2*_125_ (Table 2).

**Table 2.** Distinctive characteristics transferred with the *B. fragilis* (BF) HMW 615 *tetQ2* segment to *B. fragilis* 638R

		**Transferred Element**					
		**PSA**	**PSF**	**RM-gene shufflon**	**Shufflon EE**	**CTn341-like**	**ICE 5**
Strain	Size of transfer						
BF638R :: *tetQ2*_435_	435 KB	NO	YES	YES	YES	YES	NO
BF638R :: *tetQ2*_125_	125 KB	NO	NO	NO	YES	YES	NO
BF638R :: *tetQ2*_356_	356 KB	NO	YES	YES	YES	YES	NO
BF638R :: *tetQ2*_482_	482 KB	YES	YES	YES	YES	YES	NO

The introduction of this segment into the bacterium generated a greater diversity of R-M systems. This new R-M shufflon is immediately adjacent to the PSF operon. We had previously suggested that predation by bacteriophages is an evolutionary driving force for generation of variable polysaccharide and R-M systems in *B. fragilis* [28]. The close proximity of genes encoding R-M systems and PS biosynthetic enzymes in *B. fragilis* HMW615 means they will be co-inherited at a high frequency during conjugation. Genetic linkage between these loci suggests that part of the *tetQ2* containing segments (except for the smallest *tetQ2*_125_) could be classed as a defense island as defined by Makarova *et al*. [52] and suggests that horizontal transfer in *Bacteroides*, including the novel HGT mechanism described here, mobilizes genetic features that contribute to enhanced survival in an environment rich in bacteriophages. This transfer and homologous integration may represent a mechanism for dissemination of the multiple polysaccharide loci of *B. fragilis* as well as other genes important in resistance, virulence, host adaptation and immunogenicity.

### The *tetQ*_482_ segment is the largest chromosomal segment reported to date that was transferred between *Bacteroides* strains

The large size of the chromosomal transfer described here invites comparison to pathogenicity islands, which are generally 100 000 to 200 000 bases long and can be transferred due to proximity to a transferable element [20, 53–56]; however, in these events the pathogenicity islands integrate into the host chromosome in a site-specific manner unlike the integration of the chromosomal segments described here. Two examples of excision and mobilization of large genomic islands have been demonstrated in *Vibrio* spp. and *Mesorhizobium loti* [57, 58]. In the case of the *Vibrio* genomic islands, the experiments were conducted using mobile genetic elements (MGIs) and ICEs from *Vibrio* spp. introduced into various derivatives of *E. coli*; subsequent manipulations were all done in *E. coli* and the extent of transfer determined by the inclusion in the transferred segment of a Tn10 element inserted at various distances from the MGI. Subsequent reports of movement of large genomic islands among *Vibrio* species have been based on bioinformatic analyses of sequenced genomes [59] rather than demonstrations of actual transfer between strains. Additionally, to our knowledge, the *Mesorhizobium* 502 kb ‘symbiosis island’ is the only described genetic element larger than *tetQ2*_482_ in which mating experiments between the same or related species demonstrated mobilization, transfer and integration into the host chromosome [17, 58, 60]. In that case, the ‘symbiosis island’ containing a phage-related integrase at one end could be acquired and integrated into a tRNA gene in a nonsymbiotic *Mesorhizobium* species and is thus a mechanism by which nonsymbionts could evolve into symbionts [58]. Notably, and in contrast to the HGT event described here, integration was site-specific, required re-circularisation before integration, and the incoming DNA did not replace any of the recipient chromosome.

### Proximity of ICEs to the transferred chromosomal segments in *B. fragilis* HMW 615

**Fig. 8.**
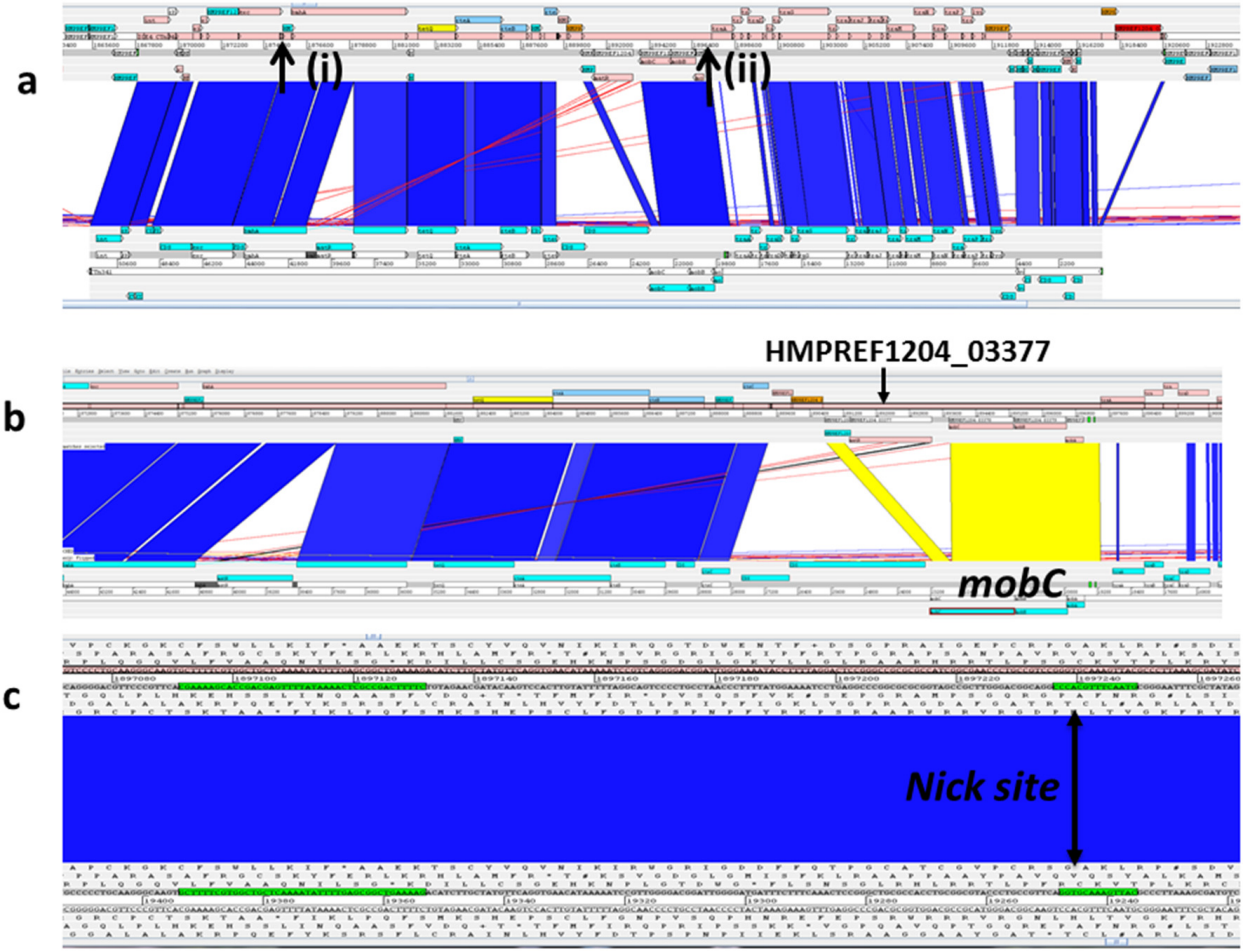
Genome comparison of the *tetQ2*_435_ within *B. fragilis* HMW615 with *B. vulgatus* CTn341 using the Artemis Comparison tool. Blue/yellow colouration between sequences indicates sequence identity, no colour indicates divergent sequence. a: Overview of complete CTn341-like region within *tetQ2*_435_ in *B. fragilis* HMW615. Arrow (i) points to the conserved RteR; arrow (ii) points to the conserved *oriT* site, inverted repeat and CTn341 nick site. b: *Mob* region. The *mobC* gene within the CTn341-like ICE in *tetQ2*_435_ is truncated by HMPREF1204_3377 (Retron-type RNA-directed DNA polymerase). c: CTn341 nick site. Note sequence conservation between CTn341 and *tetQ2*_435_.

*B. fragilis* HMW 615 has 13 regions that contain putative ICEs or ICE remnants (Fig. 1, areas indicated by pink bars) including CTnHyb, described earlier [24]. The ICEs relevant to our report include a CTn-like element transferred with *tetQ2* and the downstream ICE5, which we suggest is implicated in the transfer described in this report. A 50 000 bp segment (1867893…1920656) of the respective transferred *tetQ2*-containing segments is nearly identical to two CTns, CTn341 from *B. vulgatus* (Fig. 8) and CTnYCH46-1 from *B. fragilis* YCH46 [61, 62]. This CTn-341-like region includes the consensus ends [61, 62], the *oriT*, the nick site and *traA* to *traQ* genes (HMPREF1204_3381 to 3397) that are required to form the core channel apparatus [3, 63]; these genes are not uniformly homologous between the similar CTns. They also encode the gene for tyrosine recombinase, HMPREF1204_3354, with 98 % amino acid identity to the integrase gene in CTn341 and CTnYCH46-1 (known as IntDOT in CTnDOT [64]). An additional tyrosine recombinase gene [HMPREF1204_3285 (*xerC*) [65]] is present outside the CTn341-like region; this recombinase has only 31 % identity to the other integrases.

### Transfer of the *B. fragilis* HMW615 *tetQ2* gene was not induced by tetracycline, in contrast to many CTn-associated-*tetQ*s genes in other strains

In contrast, transfer of two *B. fragilis* conjugative transposons studied in detail, CTn341 and CTnDot, both encoding *tetQ*, are induced by tetracycline. In CTnDot, the induction by tetracycline is mediated by the action of RteABC, where RteC activates expression of excision functions, including Xis2c, subsequently leading to activation of genes required for conjugative transfer [66–73]. The *tetQ2*-chromosomal segments described here do include the *rteABC* operon (HMPREF1204_03370, 03371, and 03373, respectively) within the CTn341-like region that includes *tetQ2*. However, the gene encoding the MobC homolog, which is involved in the tetracycline induction of transfer [74], is truncated by HMPREF1204_3377 (Retron-type RNA-directed DNA polymerase) (Fig. 8). Further, CTn86 (similar to the downstream ICE 5), in contrast to most *Bacteroides* CTns, does not contain the tetracycline gene and excision is not regulated by tetracycline [75]. The estimated frequency of the transfer with tetracycline treatment remained the same as without: 1×10^−9^ (number of transconjugants [BF638R-CtnHyb]/numbers of donors [BF HMW615]). As in the mating results without tetracycline treatment, every 10th colony was *tetQ2* and the others were *tetQ1*. This strongly suggests that the *tetQ2*-chromosomal transfer events were not increased in the presence of tetracycline, which would be consistent with involvement of the tetracycline-independent CTn86-like ICE 5 in mediating the transfer.

### The HGT event mediated by the transfer of the *tetQ2*-chromosomal segments strengthens our suggestion that predation by bacteriophages is an evolutionary driving force to generate variability in polysaccharides and R-M systems in *B. fragilis*

This concept is now strengthened by our data demonstrating the close linkage between the PS, Sus-like outer membrane proteins and R-M systems with the CTn within the larger tetQ2-containing segments; this suggests that CTns actively co-mobilize genetic features that contribute to enhanced survival in an environment rich in bacteriophages and with diverse nutrient availability. Genetic linkage between these loci on these segments indicates that the R-M system genes may contribute to non-adaptive evolution of *B. fragilis* and hence could be classed as a defense island as defined by Makarova *et al*. [52]. This would also extend the definition of defense islands to include variable surface structures, such as polysaccharides and outer membrane proteins that may prevent phage adsorption. Thus, our data demonstrate that chromosomal features that contribute to enhanced survival in an environment rich in bacteriophages can be actively mobilized by ICEs on the chromosome, and raises the possibility that ICEs in *B. fragilis* have evolved to integrate in close proximity to defense islands. This event may represent a mechanism for large scale chromosomal replacement in *B. fragilis* that could confer significant advantage in adaptation.

### Conclusions

The scale of transfer of this novel HGT event has not been demonstrated before in pathogens and provides a remarkable insight into the genomic fluidity of a commensal and opportunistic pathogen. It exemplifies an efficient manner for bacteria to disseminate their pan-genome and provides a novel mechanism for adaptation to rapidly changing environments. Altering the PS surface antigenicity, surface protein variability, and R-M systems of the recipient strain will impact the highly complex and dynamic interactions of *B. fragilis* and host immune system. The scale of HGT reinforces the paradigm that pathogen characteristics are not restricted to phylogenetic boundaries but includes the genetic source of the immediate environment [14]. This idea supports the current trend toward metagenomic analysis of infectious processes that allows examination of the complex relationships in an entire population (comprising the pathogen and its immediate environment). This may be particularly important in the case of anaerobic infections that are often caused by mixed bacterial populations whose interactions may be an important part of the pathogenic process [76]. Although the selective pressures in the GI tract are the major drivers of the variability of the pan-genome in *B. fragilis*, this variability is also likely to contribute to the success of *B. fragilis* as a pathogen. It is also a potentially important mechanism for the spread of antimicrobial resistance within the GI tract microbiota.

## Supplementary Data

24 Supplementary File 1
